# One-Pot Synthesis of Silver/Zirconium Nanoparticles Using Sargassum tenerrimum for the Evaluation of Their Antibacterial and Antioxidant Activities

**DOI:** 10.7759/cureus.61779

**Published:** 2024-06-06

**Authors:** Sahith Putluru, Ramanathan Snega, P Geetha Sravanthy, Muthupandian Saravanan

**Affiliations:** 1 Department of Pharmacology, Saveetha Medical College and Hospitals, Saveetha Institute of Medical and Technical Sciences, Chennai, IND; 2 AMR and Nanotherapeutics Lab, Department of Pharmacology, Saveetha Dental College and Hospitals, Saveetha Institute of Medical and Technical Sciences, Chennai, IND

**Keywords:** silver-zirconium nanoparticles, antioxidant activity, ecofriendly, antibacterial activity, sargassum tenerimum

## Abstract

Introduction: The global health threat posed by worldwide antimicrobial resistance necessitated immediate multisectoral action by the scientific community to achieve sustainable development goals. Silver and zirconium nanoparticles (Ag/ZrO-NPs), known for their antimicrobial properties, have the potential to combat pathogens effectively, making them versatile for various applications across different fields.

Objective: This study aims to synthesize and characterize *Sargassum tenerimum-*mediated Ag/ZrO-NPs and evaluate their antioxidant and antibacterial efficacy against multidrug resistant (MDR) pathogens.

Methodology: The synthesis of Ag/ZrO-NPs using the one-pot green synthesis method was conducted and followed by using characterization techniques, namely, UV-visible spectroscopy (UV-vis), Fourier transform infrared spectroscopy (FT-IR), field emission scanning electron microscopy (FE-SEM), X-ray diffraction analysis (XRD), and energy-dispersive X-ray analysis (EDX). The antibacterial activity was assessed using the agar well diffusion method, and antioxidant activity was determined using the DPPH(2,2-diphenyl-1-picrylhydrazyl) method. Statistical analysis was analyzed using the IBM SPSS Statistics for Windows, version 21.0 (released 2012, IBM Corp., Armonk, NY).

Results: The green-synthesized Ag/ZrO-NPs exhibited a color change from dark brown to creamy white, indicating the successful reduction of the nanoparticles. UV-analysis peaks were observed at 310-330 nm, while the FT-IR analysis showed the peaks at various wavelengths, such as 648.9 cm^-1 ^(alkyne C-H bond), 1041.14 cm^-1 ^(aliphatic fluoro compounds, C-F stretch), 1382.54 cm^-1 ^(dimethyl -CH_3_), 1589.6 cm^-1 ^(primary amine, N-H bond), and 3353.8 cm^-1^ (aliphatic secondary amine, N-H stretch). The crystallinity of the nanoparticles was determined to be 59.5%, while the remaining 40.5% exhibited an amorphous structure. The SEM image revealed the spherically agglomerated structure of the nano-ranged size Ag/ZrO-NPs. The EDX analysis indicated the presence of elemental compositions Zr (16.2%), Ag (18.8%), and C (28.7%) in the green-synthesized Ag/ZrO-NPs. These nanoparticles demonstrated significant antibacterial activity against *Pseudomonas aeruginosa*, *Enterococcus faecalis,* and *Methicillin-resistant Staphylococcus aureus* (MRSA). The moderate antibacterial activity against *E. coli* showed thesignificant antioxidant activity in a dose-dependent manner.

Conclusion: The green-synthesized Ag/ZrO-NPs showed notable antibacterial and antioxidant activity. In future aspects, it may be used as a potential drug after completion of in-vivo and in-vitro studies.

## Introduction

Marine algae, being abundant and widely accessible, are recognized as a valuable natural resource. They serve as a natural reservoir of minerals, vitamins, proteins, and other bioactive substances. These substances play a crucial role as reducing agents in the synthesis of metallic nanoparticles. *Sargassum tenerimum*, a macroalgae from the *Phaeophyceae *genus of the Sargassaceae family, encompasses approximately 400 species [1]. Extensive studies have identified approximately 200 bioactive components, such as amino acids, alkaloids,carbohydrates, flavonoids, saponins, sterols, tannins,terpenoids, proteins, and phenolic compounds present in *S. tennerimum *[2]. Marine algae have primarily been utilized for industrial purposes, but their global exploitation is not yet widespread. While marine algae are commonly used as raw materials for various industrial products, their utilization in nanoparticle biosynthesis remains limited [3]. There is a scarcity of studies focusing on the synthesis of silver nanoparticles using marine algae and their potential antibacterial,antiproliferative, antifungal [4], and anticancer properties [5]. However, several marine algae show promising potential in combating various diseases, with some even undergoing clinical trials for potential use in medicine preparation. Silver nanoparticles (AgNPs) are utilized as antimicrobial agents due to their ability to combat antibiotic resistance. These nanoparticles exhibit effectiveness against both Gram-positive and Gram-negative bacteria [6]. Zirconium dioxide (ZrO_2_), also known as zirconia, is a highly stable oxide that is formed through the thermalization of zirconium compounds [7]. Depending on the specific synthesis methods employed, ZrO_2 _can exist in different crystalline phases, including monoclinic, tetragonal, and cubic structures.

Zirconium, a transition metal, exhibits corrosion resistance similar to titanium [8]. Zirconium is commonly used in dental crowns and biomaterials [9]. In addition, zirconium nanoparticles find applications in various fields, such as biosensors, anticancer treatments, antimicrobials, antioxidants, and implants [10]. Zirconium nanoparticles have also been successfully employed as drug delivery carriers for medications like penicillin, alendronate, itraconazole, and others [11]. Green synthesis methods are utilized for the synthesis of silver nanoparticles. Furthermore, hybrid nanoparticles consisting of both silver and zirconium nanoparticles can be synthesized.

The green synthesis approach involves the use of natural and environmentally friendly materials, such as reducing agents, and some of these materials can also serve as end-capping agents. This method not only minimizes energy consumption but also eliminates the need for toxic and harmful reagents [12]. Bacterial infections caused by multidrug resistance (MDR) pose an increasing global concern. Bacteria found in different body samples have demonstrated resistance to multiple classes of antimicrobial agents through various mechanisms [13]. However, wounds create a favorable environment for microbial colonization, growth, and infection due to their moisture, warmth, and nutrient availability. The most common bacterial pathogens associated with wound infection include *Staphylococcus aureus, Escherichia coli,* *Klebsiella pneumoniae*, and *Streptococcus pyogenes, Proteus species*, and *Enterococcus species* [14,15]. This study focuses on synthesizing one-pot synthesis of Ag/ZrO-NP green synthesis methods, by using the Fourier transformed infrared spectroscopy (FT-IR), X-ray diffraction (XRD), scanning electron microscopy (SEM), and energy-dispersive X-ray (EDX) spectroscopy analysis and evaluating the antibacterial activity against four antimicrobial resistance bacteria, namely, *E.coli, S.auerus, P.aeruginosa, *and *E. faecalis*) isolated from the wound infections. The evidence from this study assessed the therapeutic efficacy of Ag/ZrO-NPs in an environmentally friendly manner suggesting potential application in disease treatment and biomedical fields.

## Materials and methods

Materials and reagents

Metal oxides silver nitrate (AgNo3) and zirconium oxide (ZrO_2_) were purchased from Loba Chemie Pvt. Ltd, and the nutrient agar, Whatman no. 1 filter paper (GE HealthCare Life Sciences), and Mueller-Hinton (MH) agar were purchased from Hi-Media (Mumbai, India) and used. The bacterial cultures were obtained from the Saveetha Medical College (Saveetha Institute of Medical And Technical Sciences (SIMATS)).

Sample collection and preparation

The dried form of the *Sargassum tenerimum *(RMB-00325 - *Sargassum tenerimum J. agardh*, 1848) algal sample was obtained from Mandapam, Ramnad, and Tamil Nadu, and its authenticity was verified by the botanist Dr. N. Shiva, Assistant Professor, Department of Botany at Raja Dorasingam Government Arts College, Sivagangai, Tamil Nadu. The dried form of algal samples was cleaned to remove the impurities and dried in the shade at room temperature in an ambient environment. Using a mechanical grinder (Nanchang Kay Xin Yue Technologies Co., Jiangxi, China), 5 grams of algal extract powder was mixed with 100 ml of sterile distilled water and kept at shaker incubation for 24 hours. A 100 ml aqueous extract of algae was placed in a conical flask, while 25 ml of 25 mM silver nitrate (AgNo_3_) and 25 ml of 25 mM zirconium oxide (ZrO_2_) were added dropwise using a burette through titration. The mixture was then incubated overnight on a shaker. A color change from dark brown to creamy white was observed both initially and after 24 hours of incubation. The solution was centrifuged at 4,500 rpm for 30 minutes, and the resulting pellet was lyophilized and stored in the dried format of the nanoparticles utilized for further characterization studies and potential biomedical applications.

Characterization methods

UV-visible spectroscopy was employed to analyze the initial stage and optical properties of the nanoparticle synthesis using a Labman double-beam UV-vis spectrophotometer (LMSPUV1900S, India) and 1 cm quartz cuvettes in the 200-1000 nm range. An FT-IR analysis within the 500-3500 cm^-1^ range was conducted with a Bruker Alpha II instrument from Germany to explore the functions of various biomolecules serving as capping, reducing, and stabilizing agents in the synthesis of Ag/ZrO-NPs. XRD is commonly applied for analyzing molecular and crystal structures, a qualitative identification and resolution of active compounds, and different molecules, measurement of crystallinity, isomorphous substitution, and particle size. The phytochemical characteristics of the crystal lattice are represented by the abundance of diffraction peak values that are produced when X-ray radiation is reflected on any particles. The surface morphology of the synthesized Ag/ZrO-NPs was examined using SEM techniques with a JSM-7001F instrument by JEOL in Tokyo, Japan, operating at an accelerating voltage of 20 kV. In addition, the elemental composition of the doped nanoparticles was studied with an EDX spectrometer (OXFORD X-Plor-30/C-Swift) under a nitrogen atmosphere, heated at a rate of 10°C/min.

Antibacterial activity

The green-synthesized Ag/ZrO-NPs were tested for antibacterial activity using an agar-well diffusion process. In Muller-Hinton broth, bacterial cultures were subcultured, and the mixture was incubated for 14 hours at 37°C. Next, using a sterile cotton swab, the cultures were swabbed onto petri plates that contained Muller-Hinton agar. Agar plates were pierced with 6 mm diameter wells, and various concentrations (20, 40, 60, and 80 μg/ml) of the Ag/ZrO-NP solution were added to the wells, streptomycin was utilized as a positive control, and dimethyl sulfoxide (DMSO) was used as a negative control. After the plates were incubated, each well's zone of inhibition was assessed. The zone of inhibition (ZOI) of biosynthesized Ag/ZrO-NPs against *E. coli, P. aeruginosa, Enterococcus faecalis*, and MRSA bacterial strains was determined.

Antioxidant activity

The antioxidant was carried out by a DPPH method, which involved mixing 1 ml of Ag/ZrO-NPs at various concentrations between (10-50 μg/ml) with 1 ml of 2,2-diphenyl-1-picrylhydrazyl (DPPH) solution (1 mM in methanol). The mixture was then incubated for 30 minutes at ambient humidity in the dark. Following incubation, absorbance was measured at 520 nm. L-ascorbic acid served as a standard solution, while 0.1 mm DPPH was utilized as the control. The standard deviation was calculated using IBM SPSS Statistics for Windows, version 21.0 (released 2012, IBM Corp., Armonk, NY) after determining the mean of the triplicate results, and the antioxidant’s free radical scavenging activity was qualified as a percentage. 

Statistical analysis

The data underwent statistical analysis, and results are presented as mean values accompanied by standard deviations, based on triplicate experiments. Statistical significance was determined using one-way ANOVA with a significance level set at p < 0.05, using Microsoft Excel 2016 (Microsoft Corporation, USA).

## Results

**Figure 1:**
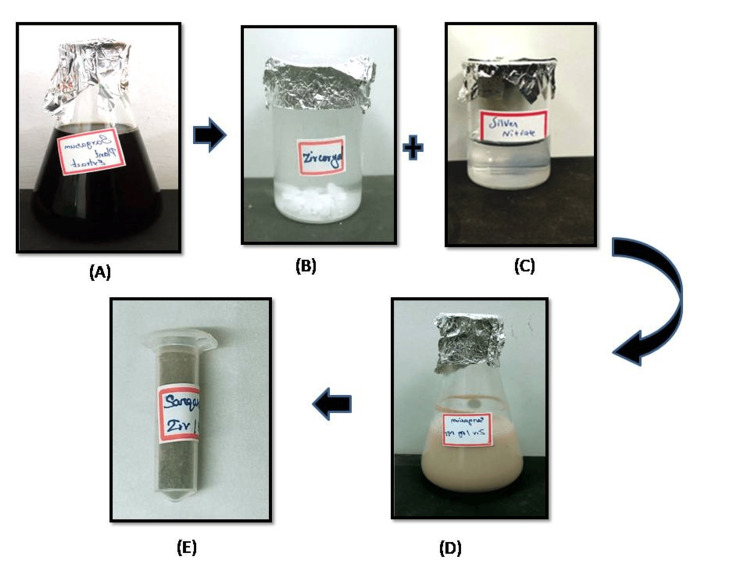
Sargassum tenerimum-mediated synthesis of Ag/ZrO-NPs. (A) Sargassum tenerimum extract. (B) 25 mM zirconium solution. (C) 25 mM silver nitrate solution. (D) After 24 hours of incubation-Ag/ZrO-NPs synthesis. (E) Ag/ZrO-NPs' dried powder form.

In this study, the green synthesis of *S. tenerimum*-mediated Ag/ZrO-NPs was utilized to synthesize by the titration method. The successful synthesis of Ag/ZrO-NPs was visually confirmed by the observed color transformation from dark brown to creamy white, as illustrated in Figure 1.

UV-visible spectroscopy analysis

**Figure 2:**
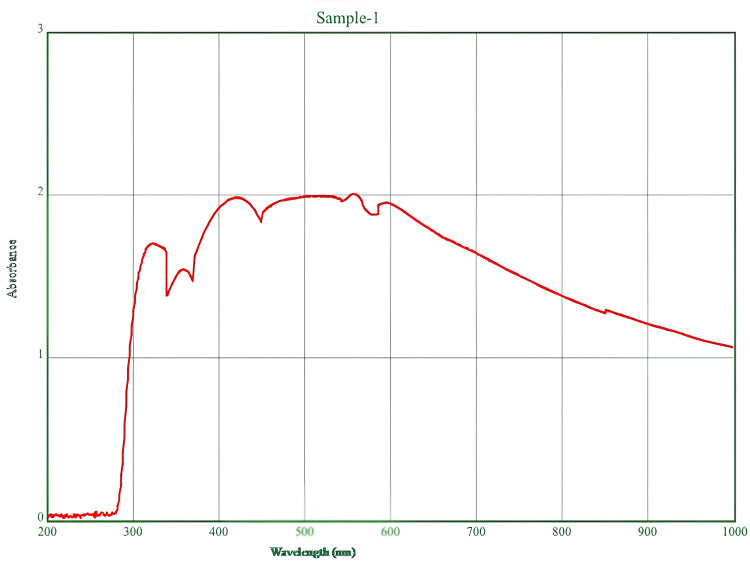
UV–vis spectroscopy analysis of the Ag/ZrO-NPs.

The main technique used to validate nanoparticles is UV-visible spectroscopy. The absorbance of the colloidal sample was measured between 200 and 1000 nm, with distilled water used as a reference. The results displayed in Figure 2 exhibited that the peaks observed in the range of 310-330 nm confirmed the presence of green-synthesized Ag/ZrO-NPs.

FT-IR analysis

**Figure 3:**
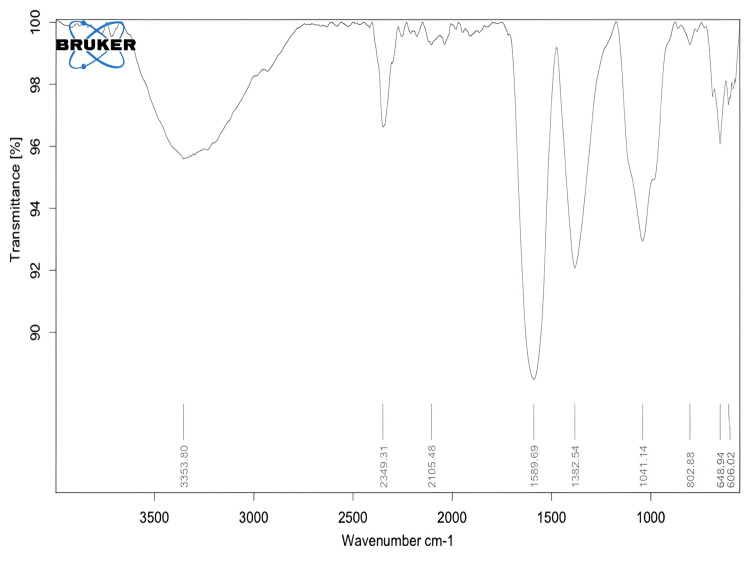
FT-IR analysis of the green-synthesized Ag/ZrO-NPs.

The green-synthesized Ag/ZrO-NPs were characterized by FT-IR spectroscopy to identify the functional groups present in the synthesized nanoparticles, and the analysis revealed the presence of more than four different functional groups in the synthesized nanoparticles. Figure 3 shows the peak values and the occurrence of compounds, such as 648.9 cm^-1^ (alkyne C-H bond), 1041.14 cm^-1 ^(aliphatic flurocompounds, C-F stretch), 1382.54 cm^-1^ (dimethyl -CH3), 1589.6 cm^-1^ (primary amine, N-H bond), and 3353.8 cm^-1 ^(aliphatic secondary amine, N-H stretch).

X-ray diffraction analysis

**Figure 4:**
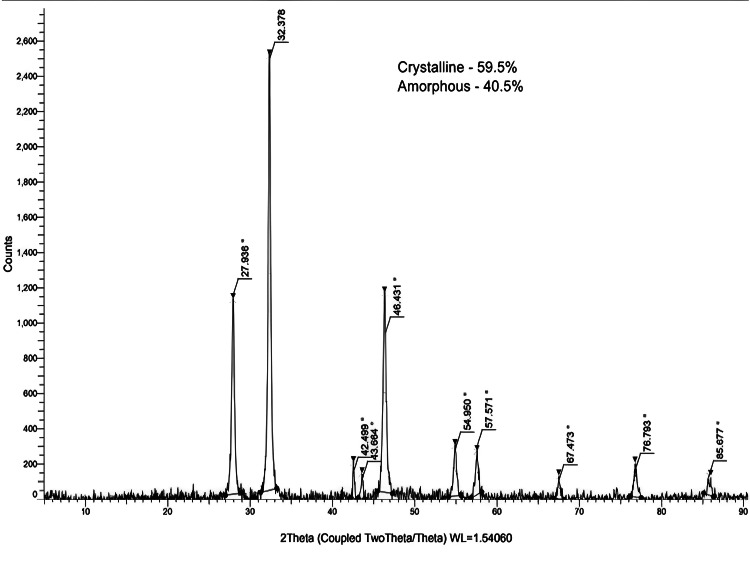
X-ray diffraction pattern of green-synthesized Ag/ZrO-NPs.

Figure 4 shows the results of the X-ray diffraction peaks at theta angles 27.9 ͦ, 32.3 ͦ, 46.4 ͦ, 54.9 ͦ, 57.5 ͦ, and 76.7 ͦ, and it revealed the crystallinity (59.5%) and amorphous (40.5%) structure.

SEM analysis

**Figure 5:**
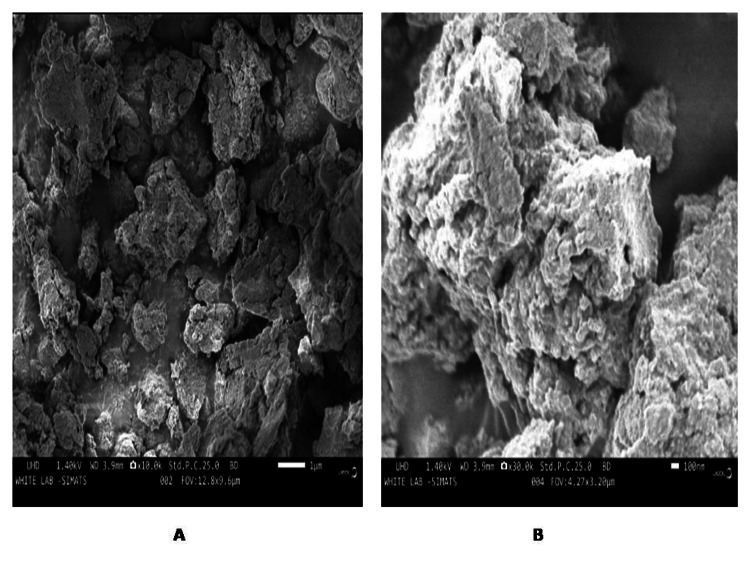
Scanning electron microscopy images of green-synthesized Ag/ZrO-NPs with different scales: A. 1 μm scale, B. 100 nm scale.

The surface morphology of the synthesized nanoparticles was examined by SEM. The different (1 μm and 100 nm) magnification ranges of the biosynthesized Ag/ZrO-NP results are shown in Figure 5. The SEM image revealed the nano-ranged size of a spherically irregular shape with the agglomerated structure of Ag/ZrO-NPs. The average size of the Ag/ZrO-NPs nanoparticles was found to be 20-50 nm.

Energy-dispersive X-ray analysis

**Figure 6:**
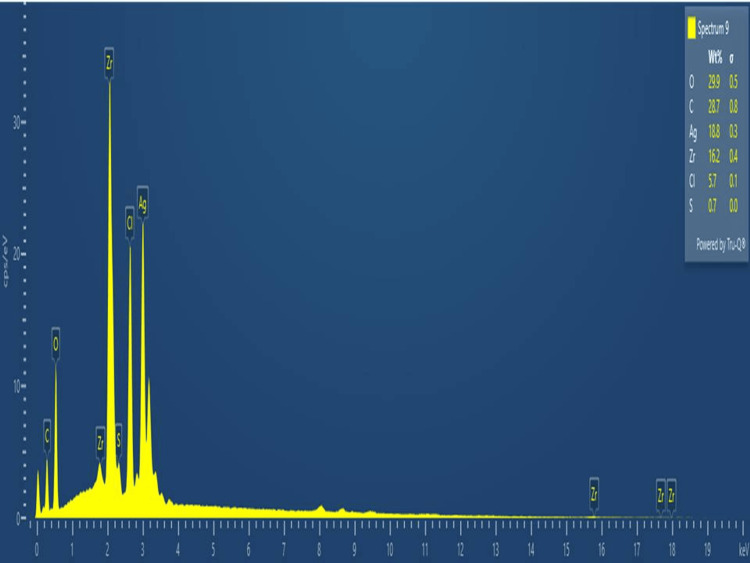
EDX analysis of green-synthesized Ag/ZrO-NPs.

The analysis was employed to determine the elemental composition and compounds present. As shown in Figure 6, the biosynthesized Ag/ZrO-NPs exhibited peaks corresponding to Zr (16.2%), Ag (18.8%), and C(28.7%). The EDX peaks observed at 1.8 keV and 2.2 keV were attributed to the presence of Zr, while the other peaks at 0.4 keV indicated the presence of carbon (C) and the peaks at 0.6 keV indicated the presence of O (oxygen). 

Antibacterial activity

**Figure 7:**
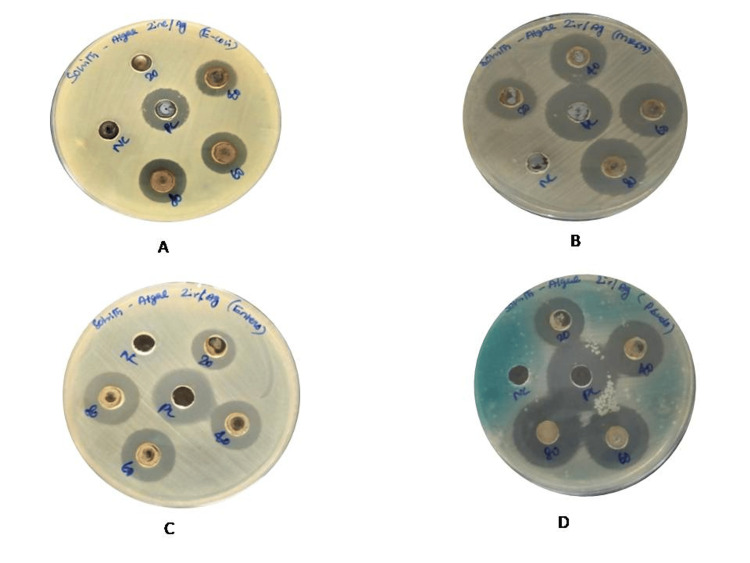
Antibacterial efficacy of green-synthesized Ag/ZrO-NPs at different concentrations: A. E. coli, B. MRSA, C. Enterococcus faecalis, D. Pseudomonas aeruginosa.

The Ag/ZrO-NPs exhibited antibacterial activity against the four bacterial isolates, namely, *E. coli*, *P. aeruginosa*, *E. faecalis*, and MRSA, at different concentrations (20, 40, 60, and 80 µg/ml) zone of inhibition, as shown in Figure 7. The antibacterial activity was notably higher against *P. aeruginosa* (20, 23, 26, and 28 mm), *E. faecalis* (17, 21, 22, and 24 mm), and MRSA (18, 22, 24, and 25 mm). However, the antibacterial activity against* E. coli* (17, 18, and 19 mm) was found to be moderate.

Antioxidant activity

**Figure 8:**
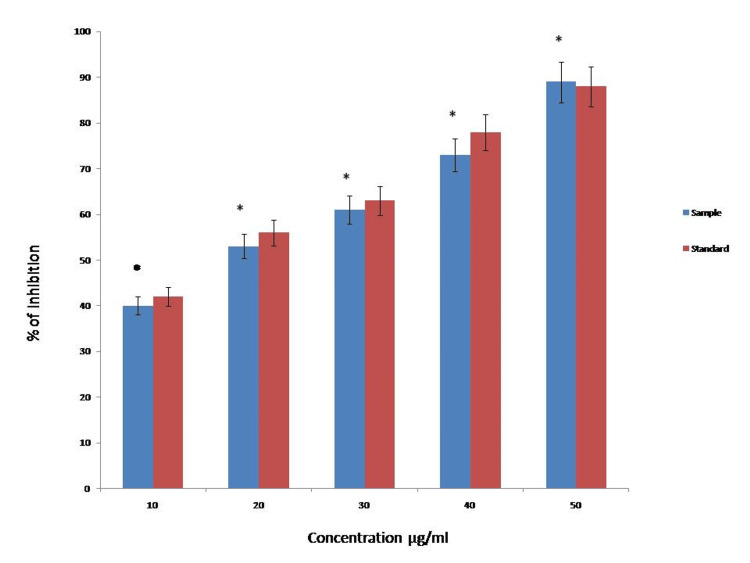
Antioxidant activity of green-synthesized Ag/ZrO-NPs compared with the standard drug. * p-value <0.05 considered as a statistical analysis.

The green-synthesized Ag/ZrO-NPs exhibited significant antioxidant activity in a concentration-dependent manner (40%, 53%, 61%, 73%, and 89%), as shown in Figure 8. A higher antioxidant activity (89%) was observed at a higher concentration of 50 µg/ml, a moderate antioxidant activity (61%) was observed at 30 µg/ml, and a lower antioxidant activity (40%) was observed at a lower concentration of 10 µg/ml.

## Discussion

In this study, the green synthesis of Ag/ZrO-NPs was conducted using the titration method (one-pot synthesis). After incubating the extract overnight, a visual color change from dark brown to creamy white was observed, indicating the formation of Ag/ZrO-NPs. Similarly, in another study, the synthesis of silver nanoparticles using *S. cinerium* resulted in a color change from yellowish green to orange, indicating the reduction of Ag+ ions [15]. Another study reported the formation of milky white precipitation, indicating the conversion of Zr(NO_3_)4.5H_2_O into nanosized ZrO_2_ colloidal particles [16]. These findings support the synthesis of AgZrO-NPs based on previous research.

In our current study, UV-visible spectroscopy was used to analyze the Ag/ZrO-NPs. The absorption peak of the Ag/ZrO-NPs was observed at 310-330 nm. The reduction of silver ions to silver nanoparticles was monitored by observing the color changes and measuring the absorbance using UV/vis spectroscopy. Silver nanoparticles typically exhibit a maximum absorption in the range of 380-450 nm due to their size-dependent optical properties [16]. The *S. cinerium* extract caused a color change from brown to reddish yellow, with a peak at 342-408 nm, indicating the formation of silver nanoparticles through the excitation of surface plasmon vibrations [17]. In another study, the ZrO_2_ NPs showed optical absorption in the range of 250-400 nm [18]. This suggests that changes in particle size and morphology did not significantly affect the optical absorption properties.

The FT-IR spectra showed more than four different functional groups present in the synthesized nanoparticles, such as 648.9 cm^-1^, 1041.14 cm^-1^, 1382.54 cm^-1^, 1589.6 cm^-1^, and 3353.8 cm^-1^. In a comparable study, the FT-IR analysis detected a band at 3350 cm^-1^, which indicates the N-H stretching vibrations of peptide linkages and hydroxyl stretch vibrations of carboxylic acid groups. This indicates the presence of polyphenols in the aqueous *Padina sp. extract* [19]. In addition, another band observed at 1637.74 cm^-1^ [20] is attributed to the vibration of the N-H primary amine group, which might be associated with the carbonyl stretch in polyphenols. Thus, the biosynthesized Ag/ZrO-NPs can be found as significant free radical scavenging potential.

The present study demonstrated the SEM analysis of Ag/ZrO-NPs, which exhibited a spherically irregular shape (agglomerated structure) with a size range of 20-50 nm. Compared to a similar study, silver nanoparticles synthesis by using *D. salina* extract showed a spherical shape with an average size of 35 nm and another study showed zirconium oxide nanoparticles mostly exhibited a sphere shape with an average size of 55 ± 5, and these particles are agglomerates of smaller particles ranging from 10 to 20 nm in size [21]. Based on the previous studies, our study confirmed that the synthesized nanoparticles are Ag/ZrO-NPs. The EDX analysis of Ag/ZrO-NPs revealed the presence of Zr (16.2%), Ag (18.8%), and C (28.7%) in the sample. In a related study, the EDX analysis of the silver nanoparticles using terrestrial fern reported the composition of Ag (16%) [22]. In addition, Sirahjul et. al reported the presence of Zr (34.1%) in the ZnO-ZrO_2_ sample. The remaining elements functioned as capping organic agents attached to the surface of the nanoparticles.

The green-synthesized Ag/ZrO-NPs exhibited notable antibacterial activity against *P. aeruginosa, E. faecalis*, MRSA, and *E. coli.* The results demonstrate a distinct bacterial response to the applied nanoparticles, showcasing their unique adhesive interactions with bacteria-targeting nanoparticles. Unlike uncoated chemically synthesized nanoparticles (AgNPs), the marine-derived biomolecules coating biologically synthesized nanoparticles improve their biological applicability, thereby enhancing their antibacterial properties [23]. Notably, biogenically synthesized Ag/ZrO-NPs can effectively bind to microbial cells and penetrate their membranes by continuously releasing nano+ ions, leading to bacterial dysfunction and eventual destruction. It has been observed that biosynthesized Ag/ZrO-NPs can significantly boost antimicrobial efficacy [24].

The antioxidant was determined by the DPPH method and the green-synthesized Ag/ZrO-NPs showed significant activity in a dose-dependent manner. The antioxidants present in the plant extract affect DPPH due to the hydrogen donation capability. DPPH, being a lipophilic radical, readily accepts electrons from the antioxidant, resulting in a color change from violet to yellow. Thus, the green-synthesized Ag/ZrO-NPs can be found as significant free radical scavenging potential [25,26].

Limitations

The current study provides important findings on the antibacterial and antioxidant properties of silver zirconium oxide nanoparticles synthesized using *S. tennerium*. Despite certain limitations, it is crucial to evaluate the durability and toxicity of these nanoparticles for their potential therapeutic uses. The emphasis on short-term observations in the study presents obstacles to gaining a comprehensive understanding of the stability and long-term effects of the nanoparticles.

## Conclusions

One-pot synthesis of Ag/ZrO-NPs with *S. tennerimum* showed significant antibacterial efficacy against MDR isolates. Furthermore, the study explored the antioxidant capabilities of these nanoparticles. The findings imply that these nanoparticles have both antibacterial and antioxidant properties, which could aid in wound healing and reduce oxidative stress. This dual functionality highlights the promise of *S. tenerimum*-mediated nanoparticle synthesis as a versatile method for infection control and healing wounds in challenging healthcare environments. Additional study of this field might end up in the development of enhanced treatments for effectively managing chronic wounds.
